# Minimizing resurgence of destructive behavior using behavioral momentum theory

**DOI:** 10.1002/jaba.499

**Published:** 2018-09-04

**Authors:** Wayne W. Fisher, Brian D. Greer, Ashley M. Fuhrman, Valdeep Saini, Christina A. Simmons

**Affiliations:** ^1^ University of Nebraska Medical Center's Munroe‐Meyer Institute

**Keywords:** behavioral momentum theory, destructive behavior, functional communication training, relapse, resurgence, translational research

## Abstract

The resurgence of destructive behavior can occur during functional communication training (FCT) if the alternative response contacts a challenge (e.g., extinction). Behavioral momentum theory (BMT) suggests that refinements to FCT could mitigate resurgence of destructive behavior during periods of extinction. Following a functional analysis and treatment with FCT, we combined three refinements to FCT (i.e., the use of a lean schedule of reinforcement for destructive behavior during baseline, a lean schedule for the alternative response during FCT, and an increase in the duration of treatment) and compared the magnitude of resurgence relative to a condition in which FCT was implemented in a traditional manner. Results suggested that the combination of these three refinements to FCT was successful in decreasing the resurgence of destructive behavior during an extinction challenge. We discuss the implications of these findings, as well as areas for future research.

Epidemiological studies and meta‐analyses have revealed that interventions based on the results of a functional analysis (FA; Iwata, Dorsey, Slifer, Bauman, & Richman [1982/1994]) are more effective than similar behavioral interventions not based on the results of an FA (Campbell, 2003; Didden, Duker, & Korzilius, 1997; Iwata, Pace, et al., 1994). One such intervention informed by the results of an FA is functional communication training (FCT), which combines differential reinforcement of alternative behavior (DRA) with extinction to teach an alternative form of communication (i.e., functional communication response [FCR]) that replaces destructive behavior. Numerous studies have shown FCT to be an effective strategy for decreasing destructive behavior reinforced by social consequences (Carr & Durand, 1985; Greer, Fisher, Saini, Owen, & Jones, 2016; Hagopian, Fisher, Sullivan, Acquisto, & LeBlanc, 1998; Kurtz et al., 2003; Matson, Dixon, & Matson, 2005; Rooker, Jessel, Kurtz, & Hagopian, 2013).

Despite its widespread effectiveness, FCT is not without limitations. For example, inadvertent lapses in treatment integrity may result in the FCR contacting unplanned and extended periods of extinction (e.g., caregivers are unable to provide the reinforcer because they are on the telephone; Fisher et al., 1993). Results of recent studies suggest that these situations may increase the likelihood of treatment relapse, wherein destructive behavior increases following successful treatment with FCT when the FCR contacts extinction (Fuhrman, Fisher, & Greer, 2016; Mace et al., 2010; Volkert, Lerman, Call, & Trosclair‐Lasserre, 2009; Wacker et al., 2011). Researchers call this form of treatment relapse *resurgence*, defined as an increase in a response previously reduced via alternative reinforcement and extinction (e.g., FCT) when alternative reinforcement terminates.

Volkert et al. (2009) observed resurgence of destructive behavior in four of five participants when the FCR contacted extinction or a thin schedule of reinforcement (i.e., abruptly transitioning from fixed‐ratio [FR] 1 to FR 12) following FCT. Mace et al. (2010) observed greater resurgence and persistence of destructive behavior following treatment with FCT plus extinction than following extinction alone. Finally, Wacker et al. (2011) showed patterns of resurgence similar to those of Volkert et al. and Mace et al. when the FCR went unreinforced during extinction probes that followed phases of FCT.

The data from these and other studies reveal a significant untoward side effect of FCT—although FCT tends to result in an immediate reduction in the level of destructive behavior, providing alternative reinforcement (e.g., for the FCR) can increase the likelihood of resurgence of destructive behavior if alternative reinforcement is later suspended (e.g., when the FCR contacts periods of extinction). It is important to note that this unfortunate side effect is not readily observable during the initial stages of FCT but can become increasingly problematic when behavior analysts attempt to generalize FCT treatment effects to caregivers in the individual's home, school, and community settings. In these contexts, caregivers may not adhere to the FCT procedures, and the newly learned FCR may go unreinforced for extended periods. Results from these initial clinical and translational investigations of resurgence following treatment with FCT suggest that it may be prudent to periodically program times throughout treatment during which reinforcement for the FCR is temporarily suspended to evaluate treatment durability (Fuhrman et al., 2016; Greer, Fisher, Romani, & Saini, 2016; Nevin & Wacker, 2013; Wacker et al., 2011).

Many researchers studying treatment relapse have employed behavioral momentum theory (BMT) as a guiding metaphor to conceptualize the behavioral processes that contribute to the resurgence of destructive behavior (Fuhrman, et al., 2016; Greer, Fisher, Romani, et al., 2016; Mace et al., 2010; Marsteller & St. Peter, 2014; Nevin & Shahan, 2011; Nevin & Wacker, 2013; Pritchard, Hoerger, & Mace, 2014; Wacker et al., 2011; 2013). In the behavioral momentum metaphor, the momentum of a response is a function of its reinforcement rate (which is equivalent to the mass of a moving object) times its baseline response rate (which is equivalent to the velocity of a moving object). In particular, an increasing number of authors have applied the quantitative models of resurgence developed by Shahan and Sweeney (2011) that predict the degree to which target responding (e.g., destructive behavior) resurges following treatments composed of extinction and alternative reinforcement (e.g., FCT, noncontingent reinforcement). Nevin and Shahan (2011) later presented the following adapted model for applied researchers, students, and practitioners:

(1) $$\frac{B_{t}}{B_{o}}=10^{\left(\frac{-t\left(c+\mathrm{dr}+pR_{a}\right)}{{\left(r+R_{a}\right)}^{0.5}}\right)}.$$

Quantitative models like those developed by Shahan and Sweeney and discussed in detail by Nevin and Shahan provide guidance on potential treatment refinements that may improve clinical outcomes by mitigating or preventing treatment relapse in the form of resurgence of destructive behavior. That is, Equation (1) makes specific and precise predictions about how the parameters of reinforcement during baseline and treatment affect the probability of the target response during each treatment session and each session in which alternative reinforcement is suspended or terminated, including whether resurgence of destructive behavior is likely to occur.

Equation (1) predicts responding at different times in extinction as a proportion of baseline responding ($\frac{B_{t}}{B_{o}}$); *B*_*t*_ represents the rate of the target response at time *t* in extinction, and *B*_*0*_ represents the mean rate of the target response during baseline. According to Equation (1), multiple variables affect the likelihood of destructive behavior when extinction is in place for both destructive behavior and the FCR following treatment with FCT. First, the parameter *c* represents the effects of terminating the contingency between destructive behavior and its reinforcer. Second, the parameter *d* represents the discriminability of the change from contingent reinforcement to extinction for destructive behavior, which Nevin, McLean, and Grace (2001) also have called the generalization decrement resulting from reinforcer omission. In Equation (1), parameter *d* scales the disruptive impact of terminating baseline reinforcement when FCT begins (with the rate of baseline reinforcement represented in Equation (1) by the parameter *r*). Third, the reductive effects of contingency termination and contingency discriminability on responding increase with the passage of time (captured by parameter *t*).

Behavioral momentum theory predicts that whereas operant extinction reduces the target response, the respondent relation between reinforcers and the prevailing context increases the persistence of the target response. For example, BMT predicts that a high rate of reinforcement for destructive behavior in a given context during baseline, captured by *r* in Equation (1), increases the persistence of that response when it contacts extinction.

It is important to note that BMT predicts that the respondent relation between reinforcers and the prevailing context increases the persistence of destructive behavior, even when the reinforcers are delivered contingent on an alternative response (as in FCT) or on a time‐based schedule (as in noncontingent reinforcement). That is, alternative reinforcement (e.g., delivered contingent on an FCR) acts to suppress destructive behavior during treatment, but it also may strengthen the persistence of destructive behavior through the respondent pairings of reinforcers and the stimulus context. However, this strengthening effect becomes apparent only when alternative reinforcement ceases and its suppressive effects are therefore no longer in place. Both basic studies involving nonhuman species and translational studies involving individuals with developmental disabilities have demonstrated this strengthening effect of alternative reinforcement (e.g., Mace et al., 2010; Nevin, Tota, Torquato, & Shull, 1990). Equation (1) captures the effects of alternative reinforcement with the parameter *R*_*a*_.

To summarize, Equation (1) predicts greater resurgence following (a) relatively higher rates of reinforcement (*r*) in baseline, (b) relatively higher rates of alternative reinforcement (*R*_*a*_) in treatment, (c) short exposures to treatment (*t*), and (d) less discriminable transitions from reinforcement to extinction (*d*). These same predictions also imply procedural refinements to FCT that should minimize the resurgence of destructive behavior during periods when the FCR contacts either unplanned periods of extinction (e.g., when a parent is busy and unable to reinforce the child's FCRs) or planned periods of extinction (e.g., when an experimenter introduces an extinction challenge). For example, Equation (1) predicts greater resurgence during an extinction challenge if destructive behavior results in a high reinforcement rate in baseline (i.e., a large value of *r*).

In clinical practice, destructive behavior is often associated with a high rate of reinforcement in baseline because clinicians typically provide the functional reinforcer for destructive behavior on an FR 1 schedule to mimic the contingencies programmed in the corresponding test condition of the FA. Equation (1) suggests that this practice of arranging a dense reinforcement schedule in baseline will increase the likelihood of observing resurgence if the FCR later results in extinction. Therefore, one potential refinement of FCT based on Equation (1) would be to provide a lean schedule of reinforcement for destructive behavior during baseline (i.e., reducing the value of *r*).

Another refinement of FCT suggested by Equation (1) involves the rate of alternative reinforcement delivered for the FCR during FCT. Equation (1) predicts greater resurgence when the FCR produces a high rate of alternative reinforcement (i.e., a large value of *R*_*a*_). In clinical practice, FCT often begins with an FR 1 schedule in which each instance of the FCR results in the delivery of the functional reinforcer. Such dense reinforcement schedules produce a high rate of alternative reinforcement, which according to Equation (1) increases the likelihood of resurgence. Thus, an additional refinement of FCT would be to provide reinforcement for the FCR on a lean schedule of reinforcement during FCT (i.e., reducing the value of *R*_*a*_).

Equation (1) also predicts differential levels of resurgence following short and long exposures to FCT, with greater resurgence following treatments implemented in a fewer number of sessions or shorter amount of time (i.e., a small value of *t*). In clinical practice, behavior analysts may too quickly assess for the generalization of FCT treatment effects, doing so once the treatment appears effective in the context in which it was first implemented. Therefore, another refinement of FCT would be to provide a longer exposure to (or greater dosage of) treatment than standard of care would otherwise suggest (i.e., increasing the value of *t*).

To summarize, Equation (1) identifies at least three refinements to FCT that should each reduce the likelihood of resurgence of destructive behavior if the newly acquired FCR contacts periods of extinction. Programming a lean schedule of reinforcement in baseline and throughout FCT, as well as increasing the dosage of FCT by conducting additional sessions of treatment should minimize the likelihood of resurgence. However, Equation (1) further suggests that combining these three refinements within a single evaluation of FCT (i.e., arranging a low rate of reinforcement in baseline followed by a lengthy exposure to FCT implemented with a low rate of alternative reinforcement) should result in less resurgence than would any of these refinements implemented alone. In the present study, we combined these three refinements to FCT and compared the degree to which destructive behavior resurged following FCT procedures with and without these three refinements.

## GENERAL METHOD

Four individuals referred to a university‐based severe behavior disorders clinic participated. Erica, a 16‐year‐old girl, Corey, a 3‐year‐old boy, Jaden, an 8‐year‐old boy, and Derek, a 7‐year‐old boy, each were diagnosed with autism spectrum disorder (ASD). Erica also carried the diagnosis of attention deficit hyperactivity disorder (ADHD). All participants engaged in self‐injurious behavior (SIB) and aggression. Corey, Jaden, and Derek also engaged in property destruction. All participants communicated using utterances of one‐to‐four words. We conducted all study procedures under the oversight of a pediatrics institutional review board and followed the safety precautions described by Betz and Fisher (2011) to protect the safety of the participants.

### *Settings and Materials*

All sessions took place in 3‐m by 3‐m therapy rooms equipped with a two‐way intercom system and a one‐way observation window. Therapy rooms for Corey, Jaden, and Derek contained padding on the walls and floors to minimize the risk of injury associated with their SIB. Furniture (e.g., table, chairs, desk) remained present in the therapy rooms for all participants except Derek. Sessions for Derek occurred in an empty therapy room due to the risk of injury associated with his topography of SIB (i.e., slamming knees and elbows against hard surfaces).

### *Response Measurement, Interobserver Agreement, and Blinding Procedures*

Trained observers collected data on laptop computers behind the observation window. We collected frequency data on SIB, aggression, property destruction, and the FCR. *Self‐injurious behavior* included self‐biting, body slamming, self‐hitting, self‐scratching, and head banging. *Aggression* included hitting, kicking, pushing, pinching, scratching, or throwing objects at the therapist. *Property destruction* included hitting or kicking furniture or the walls or floor of the therapy room, throwing objects not meant to be thrown (but not at the therapist), tearing one's own clothing, swiping materials, and turning over furniture. *Functional communication responses* consisted of the individual touching (Erica) or exchanging (Corey, Jaden, and Derek) an index‐sized card that contained a picture of the child consuming their functional reinforcer (i.e., the FCR card).

We obtained interobserver agreement (IOA) by having a second independent observer collect data simultaneously with the primary data collector on a minimum of 26% of sessions. For the experiment proper, we required the second observer to be blind to the study purpose and hypotheses for a minimum of 27% of the sessions for which we collected IOA. We divided each session into 10‐s intervals and scored an agreement for each interval in which both observers measured the same number of responses (i.e., exact agreement). We then summed the number of agreement intervals and divided by the number of agreement intervals plus disagreement intervals. Finally, we converted each quotient to a percentage. We calculated IOA on at least 33% of sessions of each participant's functional analysis and initial FCT evaluation. Coefficients averaged 98% (range, 67%‐100%) for Erica, 99% (range, 80%‐100%) for Corey, 99% (range, 87%‐100%) for Jaden, and 97% (range, 50%‐100%) for Derek. We calculated IOA on at least 26% of sessions for each participant in the experiment proper. Coefficients averaged 97% (range, 73%‐100%) for Erica, 98% (range, 72%‐100%) for Corey, 99% (range, 67%‐100%) for Jaden, and 95% (range, 50%‐100%) for Derek.

### *Functional Analysis and Initial Evaluation of Functional Communication Training*

#### *Functional analysis*

We conducted FAs of each participant's destructive behavior to identify its maintaining variables using procedures similar to those described by Iwata, Dorsey, et al. (1982/1994). Our procedures differed from Iwata, Dorsey, et al. in that (a) we did not include avoidance contingencies in the escape condition, (b) we included a tangible (test) condition (Day, Rea, Schussler, Larsen, & Johnson, 1988), (c) we equated reinforcer‐access durations across test conditions (Fisher, Piazza, & Chiang, 1996), and (d) we began each FA by screening for the presence of automatically reinforced destructive behavior (Querim et al., 2013). In some test and control conditions of the FA, we also used the results of a paired‐stimulus preference assessment to identify the stimuli used in those conditions (Fisher et al., 1992). A trained therapist conducted all FA sessions with the exception that Corey's mother and caregiver conducted portions of his FA. Each FA session lasted 5 min.

In the alone condition (Erica only), the participant remained alone in the therapy room without any toys or materials. Destructive behavior produced no programmed consequence. In the ignore condition (Corey, Jaden, and Derek), the participant and therapist remained in the therapy room together without any toys or materials. The therapist ignored all instances of destructive and appropriate behavior throughout the session. Prior to the attention condition, the therapist provided the participant with 1‐min access to physical and vocal attention (e.g., playing a game and providing high fives). The attention condition began with the therapist withdrawing and diverting their attention to a magazine. The participant retained free access to a low‐preference toy throughout the attention condition, and destructive behavior resulted in the therapist returning their attention to the child for 20 s. In the escape condition, the therapist delivered academic or household‐related demands using a least‐to‐most (i.e., verbal, model, physical) prompting hierarchy. Destructive behavior produced a 20‐s break from instructions in which the therapist removed all instructional materials. Prior to the tangible condition, the therapist provided 1‐min access to a high‐preference toy, and the tangible condition began with the therapist restricting access to that toy. The therapist redelivered the high‐preference toy for 20 s contingent on destructive behavior. In the toy‐play condition, the participant had continuous access to a high‐preference toy, and the therapist provided physical and vocal attention at least every 30 s. The therapist provided no programmed consequences for destructive behavior.

#### *Initial evaluation of FCT*

We conducted an initial evaluation of FCT using a reversal design to determine the effectiveness of FCT as a treatment for each participant's destructive behavior following the completion of each participant's FA. We treated the tangible function of Erica's, Jaden's, and Derek's destructive behavior and the attention function of Corey's destructive behavior in this and all subsequent implementations of FCT.

#### *Baseline*

The baseline condition of the initial FCT evaluation was identical to the tangible (Erica, Jaden, and Derek) or attention (Corey) condition of the FA. Sessions lasted 5 min.

#### *Pretraining (data not displayed)*

Following the initial baseline phase, we used a progressive‐prompt delay (0 s, 2 s, 5 s, 10 s) to teach each participant to emit the FCR to gain access to the reinforcer maintaining destructive behavior. Instances of the destructive response resulted in no programmed consequences (i.e., extinction). Each 10‐trial session consisted of the therapist presenting the establishing operation for destructive behavior (e.g., by withholding the preferred toy or attention), prompting the FCR using physical guidance if necessary, and delivering the functional reinforcer for 20 s on an FR 1 schedule. The FCR for all participants consisted of touching (Erica) or exchanging (Corey, Jaden, and Derek) a picture card that contained an image of the participant consuming the functional reinforcer. Delays to the therapist prompting the FCR increased every two consecutive sessions with no destructive behavior. Pretraining terminated following two consecutive sessions with no destructive behavior and independent FCRs in 80% or greater of trials. We used a 3‐s changeover delay (COD; Herrnstein, 1961) to prevent adventitious reinforcement of destructive behavior. If destructive behavior occurred within 3 s of the participant emitting the FCR, the therapist withheld the reinforcer until the participant emitted another FCR without destructive behavior occurring within 3 s. Pretraining session durations varied depending on the prompt delay, as well as on the presence and efficiency of independent FCRs.

#### *FCT*

We implemented FCT using procedures identical to pretraining except that we discontinued all prompts to emit the FCR, and sessions lasted 5 min.

### *Results*

**Figure 1:**
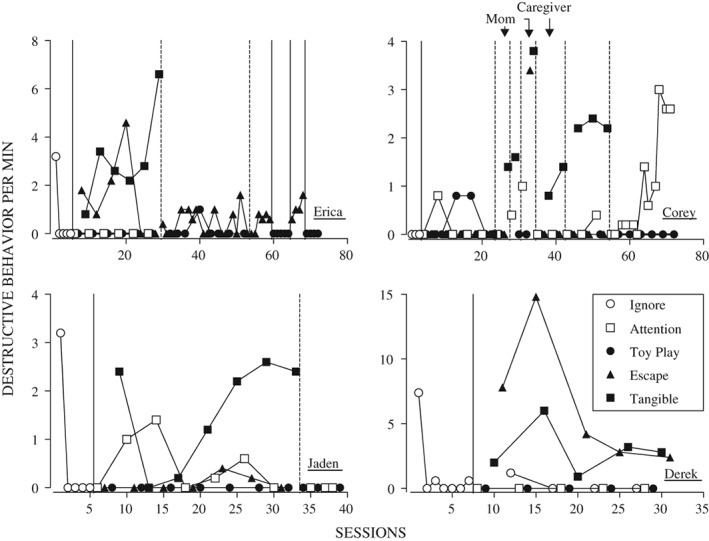
Functional‐analysis results for Erica, Corey, Jaden, and Derek. A therapist conducted all sessions with Corey other than in those phases labeled otherwise.

Erica (top left panel of Figure 1) displayed no destructive behavior in the final four consecutive‐ignore sessions. Erica then engaged in elevated rates of destructive behavior during the tangible condition and near‐zero rates in the attention and toy‐play conditions. Because we observed variable rates of destructive behavior across sessions of the escape condition, we conducted a pairwise analysis with the escape and toy‐play conditions. Variability persisted following this change in experimental design, at which point we conducted a reversal design between the escape and toy‐play conditions to better determine whether escape from demands reinforced Erica's destructive behavior. Erica emitted higher rates of destructive behavior in the escape condition relative to the toy‐play condition in the reversal design. Erica's FA results suggest that access to preferred tangible items and escape from demands reinforced her destructive behavior. We treated the tangible function of Erica's destructive behavior using the procedures described in this paper and later treated her escape function using a separate protocol.

Corey (top right panel of Figure 1) displayed low, variable rates of destructive behavior across only the attention and toy‐play conditions of the multielement FA. Between FA sessions, however, therapists observed that Corey frequently engaged in destructive behavior with his mother and caregiver. Therefore, we had each of these individuals serve as therapist in subsequent FA sessions and sequenced those sessions based on the availability of each individual. We observed consistently elevated rates of destructive behavior in the tangible condition across both Corey's mother and caregiver. We then returned to the therapist‐conducted multielement FA and replicated this same pattern of responding. Due to the variable rates of Corey's destructive behavior across individuals in the attention condition, we conducted a pairwise analysis with the attention and toy‐play conditions and observed an increasing trend in the rates of destructive behavior in the attention condition and no instances in the toy‐play condition. Corey's FA data suggest that access to preferred tangible items and adult attention reinforced his destructive behavior. We treated the attention function of Corey's destructive behavior using the procedures described in this paper and addressed his tangible function using a separate protocol. We targeted the attention function of Corey's destructive behavior to increase the variety of functions targeted across participants.

Jaden (bottom left panel of Figure 1) displayed no destructive behavior in the final four consecutive‐ignore sessions preceding his multielement FA. Thereafter, Jaden engaged in destructive behavior during the tangible, attention, and escape conditions with consistently elevated rates in only the final three sessions of the tangible condition. Jaden displayed no destructive behavior in the toy‐play condition. A pairwise analysis between attention and toy‐play conditions produced no destructive behavior. Jaden's FA results suggest that access to preferred tangible items maintained his destructive behavior.

Derek (bottom right panel of Figure 1) emitted near‐zero rates of destructive behavior in the final six consecutive‐ignore sessions that preceded his multielement FA. Derek's multielement FA produced consistently elevated rates of destructive behavior in both the tangible and escape conditions and no instances in the toy‐play condition. Derek's FA results suggest that access to both preferred tangible items and escape from demands maintained his destructive behavior. We treated the tangible function of Derek's destructive behavior using the procedures described in this paper and addressed his escape function using a separate protocol. We targeted the tangible function of Derek's destructive behavior due to observing more consistent rates of responding in this condition relative to the escape condition.

**Figure 2:**
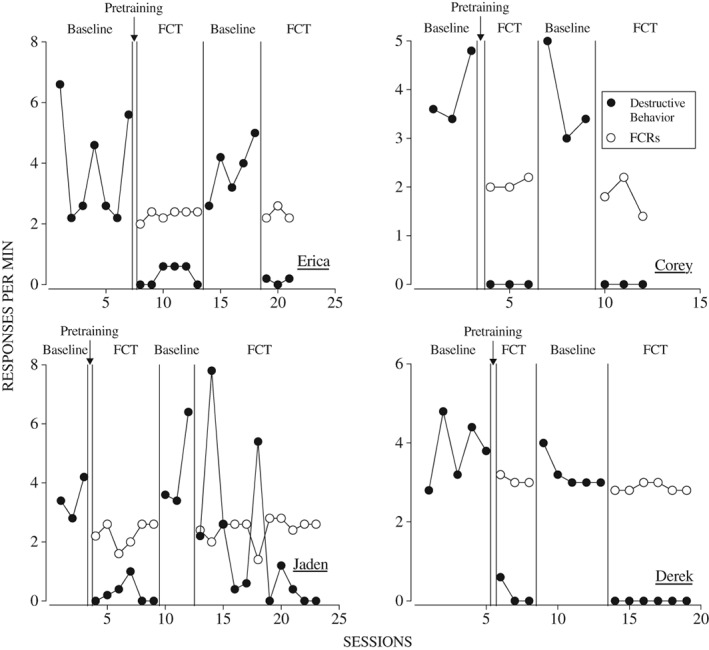
Results of the initial evaluation of functional communication training (FCT) for Erica, Corey, Jaden, and Derek.

All four participants displayed elevated rates of destructive behavior prior to FCT pretraining. During FCT pretraining (not displayed in Figure 2), all participants engaged in low rates of destructive behavior and increasingly high rates of independent FCRs. Pretraining lasted 23 sessions for Erica, 9 sessions for Corey, 14 sessions for Jaden, and 17 sessions for Derek. Following pretraining, we observed marked reductions in rates of destructive behavior for all four participants and high rates of the FCR during FCT; these effects were then replicated.

## BMT‐INFORMED REFINEMENTS TO FCT

We evaluated the combined effects of reinforcement rate (during baseline and treatment) and the dosage of treatment on the resurgence of destructive behavior by programming a lean schedule of reinforcement for destructive behavior in baseline, a lean schedule of reinforcement for the FCR during FCT, and triple the number of FCT sessions in the test condition. We tested for resurgence within the context of a multielement ABC resurgence paradigm in which we reinforced destructive behavior in baseline (Phase A), placed destructive behavior on extinction and reinforced the FCR during FCT (Phase B), and then arranged extinction for both the destructive behavior and the FCR in the final phase (Phase C). Across these three phases, we programmed two separate conditions (i.e., lean–long [test condition], dense–short [control condition]). In the lean–long condition, we delivered a lean schedule of reinforcement across baseline and FCT phases, and we provided a longer exposure to FCT (i.e., a larger dose) than in the dense–short condition. By contrast, we delivered a dense schedule of reinforcement across baseline and FCT phases in the dense–short condition, and we provided a shorter exposure to FCT (i.e., a smaller dose) than in the lean–long condition. To facilitate discrimination between these two FCT conditions, we assigned each condition a unique therapist and unique color‐correlated stimuli. For example, in one condition (e.g., lean–long), we included blue light filters and a blue FCR card, and Therapist A conducted sessions wearing a blue scrub top. In the other condition (dense–short), we included yellow light filters and a yellow FCR card, and Therapist B conducted sessions wearing a yellow scrub top (Conners et al., 2000; Mace et al., 2010).

### *Progressive‐Interval Assessment*

We conducted a progressive‐interval assessment (PIA) similar to that described by Findley (1958) with each participant to empirically identify an optimally lean schedule of reinforcement for use in the lean–long condition that did not extinguish responding or result in adverse effects (e.g., bursts of destructive behavior; Knutson & Kleinknecht, 1970). We also used the results of the PIA to select the relatively dense reinforcement schedule for use in the dense–short condition. The results of the PIA for all participants suggested two variable‐interval (VI) schedules of reinforcement that remained constant across baseline and FCT phases yet differed across lean–long and dense–short conditions. We used the constant‐probability distribution described by Fleshler and Hoffman (1962) for all VI schedules.

Each trial of the PIA began with the therapist presenting the establishing operation for destructive behavior (i.e., by removing a preferred item [Erika, Jaden, and Derek] or by withdrawing attention [Corey]) and then terminating the establishing operation after the first instance of destructive behavior that followed expiration of the current fixed interval (FI). Exposure to the establishing operation increased following two trials at each of the following FIs: 2 s, 4 s, 8 s, 10 s, 15 s, 20 s, 30 s, 45 s, 65 s, 90 s, 120 s, 150 s, and 180 s. The PIA terminated following (a) a burst of destructive behavior (i.e., three instances within 5 s) or negative emotional responding (i.e., 5 s of continuous negative vocalizations or crying) or (b) after the second trial at FI 180 s, whichever came first. The PIA lasted one continuous session and terminated due to a burst of destructive behavior for each participant.

With the first two participants who experienced the PIA (Erica and Jaden), we used the PIA results to select the leanest schedule of reinforcement that did not evoke untoward side effects (i.e., a burst of destructive behavior or negative emotional responding). For example, if untoward side effects, as defined above, occurred at the FI 45‐s schedule during the PIA, we selected the preceding PIA schedule (i.e., the FI 30‐s schedule) for use in the lean–long condition (i.e., a VI 30‐s schedule). We used FI schedules during the PIA to promote schedule discrimination and VI schedules for the baselines to promote relatively high and steady responding during baseline.

After selecting the baseline schedule for the lean–long condition, we then divided this lean reinforcement schedule by 4.5 to determine the dense VI schedule for use in the dense–short condition. This resulted in greater than a fourfold difference in the programmed rates of reinforcement between the lean–long and dense–short conditions in the baseline and FCT phases (e.g., Shahan, Magee, & Dobberstein, 2003).

Unfortunately, this process of deriving VI schedules resulted in the premature extinguishing of Erica's destructive behavior across both the lean–long and dense–short conditions in the initial baseline (not depicted). To maintain Erica's responding, we reduced the reinforcement schedules in both conditions by half and conducted a new baseline phase. To avoid this same problem with subsequent participants (Corey and Derek), we supplemented our PIA results with data on the average latency to destructive behavior following each withdrawal of the reinforcer during the last five baseline sessions from the initial FCT evaluation. Thus, we selected the PIA‐equivalent schedule that was just shorter than the average latency to destructive behavior for use as the VI schedule in the dense–short condition. For example, if the latency to destructive behavior averaged 24 s across the preceding five FCT baseline sessions, we hypothesized that a slightly denser VI schedule (e.g., VI 20 s) should circumvent most destructive behavior. We compared the results we obtained from this latency analysis with the results we obtained from the PIA for each subsequent participant and selected the denser of the two obtained durations as the reinforcement schedule for the dense–short condition. We then multiplied this reinforcement schedule by 4.5 to determine the lean reinforcement schedule for the lean–long condition. In addition, we made individual adjustments to the reinforcement schedules if the trend in response rates showed a steady decline during baseline or if obtained reinforcement rates differed greatly from those programmed.

### *Baseline*

We conducted baseline sessions using procedures identical to those in the functional analysis and initial FCT evaluation, except (a) we derived and then implemented the reinforcement schedules for destructive behavior using the procedures described above, and (b) sessions lasted 10 min. Baseline terminated when (a) there were at least five sessions in both conditions, (b) the trend for each baseline was flat or in the direction opposite the goal for treatment, and (c) the standard deviations of responding in the last five baseline sessions of each condition were no more than 50% of their mean.

#### *Lean–Long*

We delivered the functional reinforcer for destructive behavior according to a lean VI schedule of reinforcement. Based on the PIA or latency analysis described above, we selected the following individualized lean schedules: Erica, VI 23 s; Corey, VI 23 s; Jaden, VI 90 s; and Derek, VI 14 s. Equation (1) predicts that programming a lean reinforcement schedule in baseline and in an extended treatment in which extinction is arranged for destructive behavior should decrease the likelihood of resurgence during a subsequent extinction challenge.

#### *Dense–Short*

We delivered the functional reinforcer for destructive behavior according to a VI schedule of reinforcement that was 4.5 times as dense as the lean schedule described above. Using this multiplication factor, we selected the following individualized dense schedules: Erica, VI 5 s; Corey, VI 5 s; Jaden, VI 20 s; and Derek, VI 2 s.

### *FCT*

We implemented FCT using the same procedures from the initial FCT evaluation, except (a) the same VI schedules most recently in place for destructive behavior in the preceding baseline phase were arranged for FCRs, and (b) sessions lasted 10 min. Therapists placed destructive behavior on extinction across all FCT conditions. Additionally, we conducted three sessions of the lean–long condition for every one session of the dense–short condition to increase the dosage of FCT in the lean–long condition. Thus, we quasirandomly ordered sessions in blocks of four (i.e., one dense–short and three lean–long sessions) such that no more than two dense–short and no more than six lean–long sessions occurred consecutively. The FCT phase terminated following two consecutive sessions in each condition in which destructive behavior was at or below an 85% reduction from average responding in the corresponding baseline condition.

### *Extinction Challenge*

We conducted identical extinction‐challenge sessions across the lean–long and dense–short conditions. During the extinction challenge, we placed both the FCR and destructive behavior on extinction, and the therapist delivered 20‐s access to the functional reinforcer according to a tandem variable‐time (VT) 200‐s schedule with a 3‐s differential reinforcement of other behavior (DRO) schedule to prevent adventitious reinforcement of destructive behavior and the FCR. Thus, if destructive behavior or the FCR occurred within 3 s of the scheduled tandem VT‐DRO delivery, the therapist withheld the scheduled reinforcer until the participant had not emitted destructive behavior or the FCR for 3 s. We included these occasional time‐based reinforcer deliveries to decrease the discriminability between the treatment and extinction phases (see Nevin & Shahan's 2011 discussion of Koegel & Rincover, 1977; pp. 883‐884).

### *Results and Discussion*

**Figure 3:**
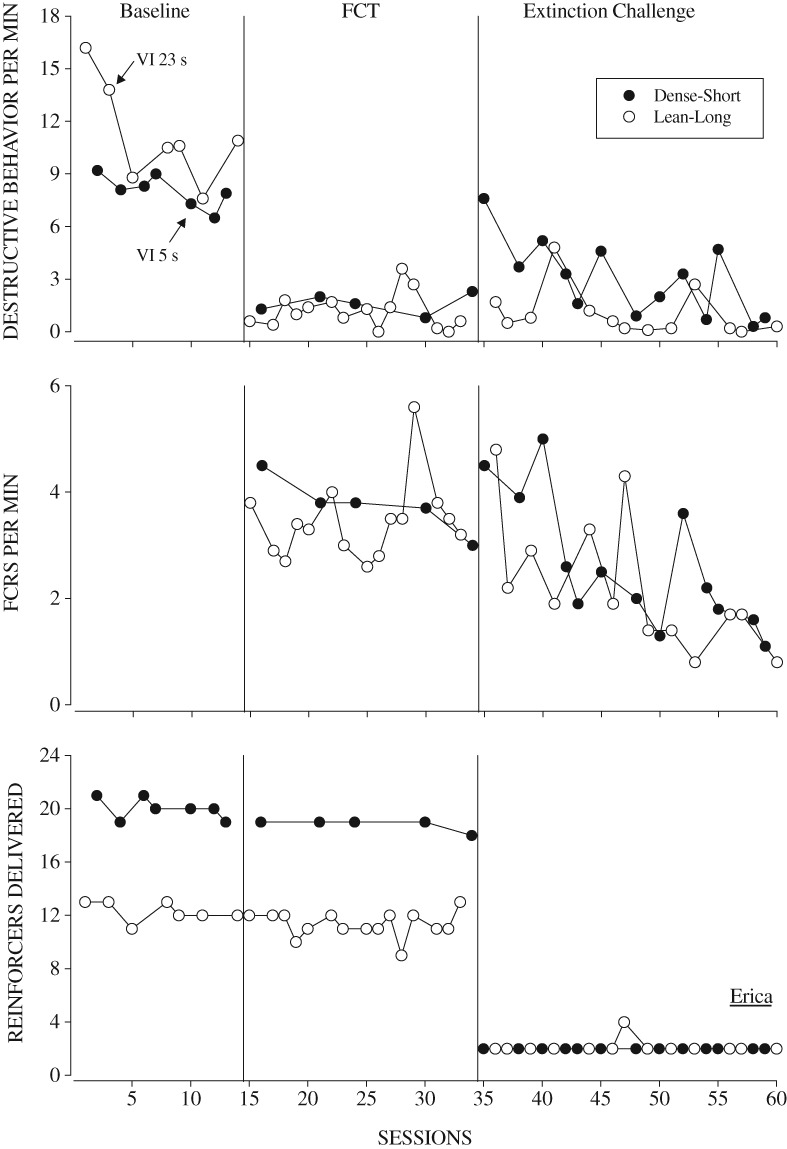
Erica's results during the dense–short and lean–long conditions across baseline, functional communication training (FCT), and extinction phases.

Figure 3 displays the rates of destructive behavior (top panel) and FCRs (middle panel), as well as the number of reinforcers delivered (bottom panel) during the lean–long (i.e., VI 23‐s) and dense–short (i.e., VI 5‐s) conditions for Erica. Erica engaged in high rates of destructive behavior across both conditions in baseline and experienced a greater number of reinforcers during the dense–short condition (*M* = 20.0 reinforcers per session) than in the lean–long condition (*M* = 12.3 reinforcers per session). Rates of destructive behavior decreased in both the lean–long and dense–short conditions during FCT, with rates of destructive behavior being slightly higher in the dense–short condition (*M* = 1.6 responses per min [RPM]) than in the lean–long condition (*M* = 1.2 RPM). Rates of the FCR maintained across conditions at similar rates in the lean–long (*M* = 3.4 RPM) and dense–short (*M* = 3.8 RPM) conditions. We observed greater variability of both destructive behavior and FCRs during the lean–long condition of the FCT phase. Reinforcer deliveries maintained at similar levels during the FCT phase as in baseline, with higher reinforcer deliveries in the dense–short condition (*M* = 18.8 reinforcers per session) relative to those in the lean–long condition (*M* = 11.3 reinforcers per session). Despite delivering more reinforcers per session in the dense–short condition, the total number of reinforcers delivered in the dense–short condition of FCT (94 total reinforcers) was fewer than the total number of reinforcers delivered in the lean–long condition of FCT (170 total reinforcers). During the extinction challenge, we observed greater resurgence of destructive behavior in the dense–short condition (*M* = 3.0 RPM) relative to the lean–long condition (*M* = 1.0 RPM). Erica's use of the FCR declined across conditions of the extinction challenge, and the tandem VT‐DRO schedule produced consistent rates of reinforcement across conditions (*Ms* = 2.2 and 2.0 reinforcers per session in lean–long and dense–short conditions, respectively).

**Figure 4:**
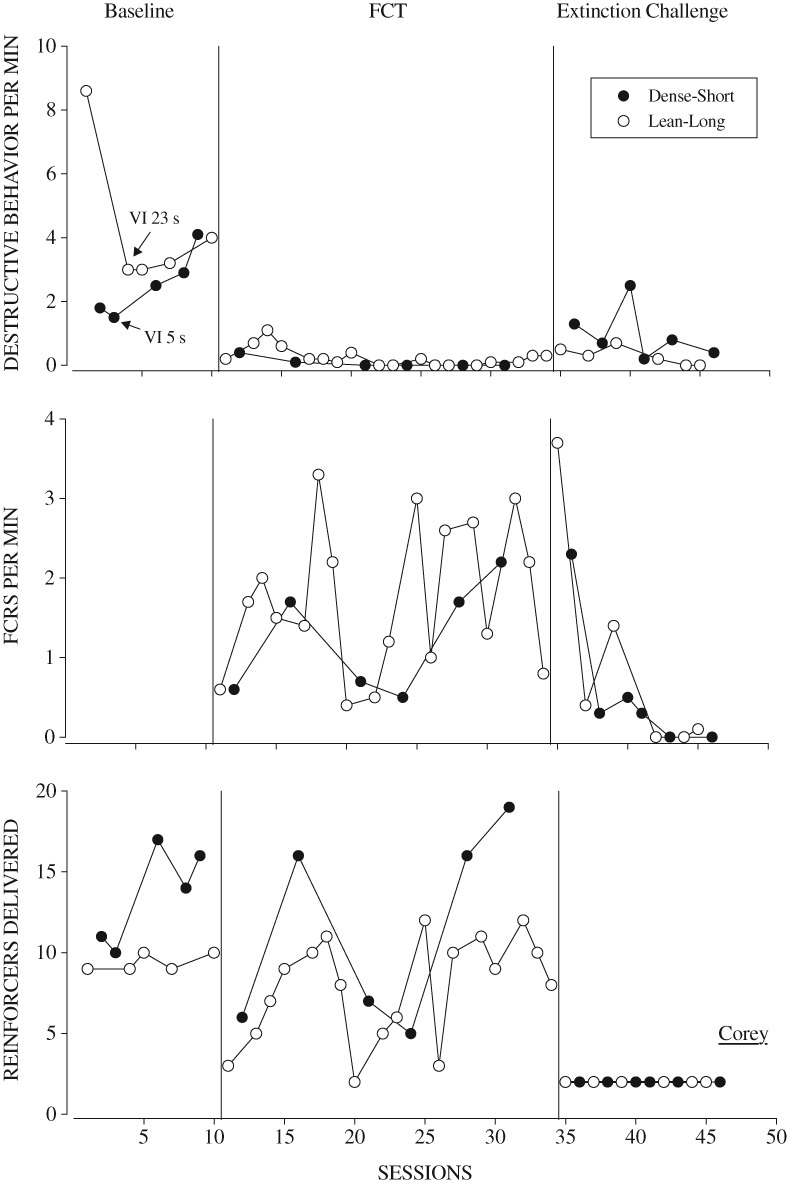
Corey's results during the dense–short and lean–long conditions across baseline, functional communication training (FCT), and extinction phases.

Figure 4 displays Corey's results. Corey engaged in elevated and increasing rates of destructive behavior over the last four baseline sessions during both conditions and experienced a greater number of reinforcers during the dense–short condition (*M* = 13.6 reinforcers per session) than in the lean–long condition (*M* = 9.4 reinforcers per session) of baseline. Like Erica's results, FCT rapidly suppressed Corey's high rates of destructive behavior in both the lean–long and dense–short conditions, with a slightly higher rate of destructive behavior in the lean–long condition (*M* = 0.3 RPM) relative to the dense–short condition (*M* = 0.1 RPM). Corey emitted moderate to high rates of the FCR across both conditions of FCT, with a similar rate of FCRs in the lean–long (*M* = 1.7 RPM) and dense–short (*M* = 1.2 RPM) conditions. Corey experienced slightly more reinforcers in the dense–short condition of FCT (*M* = 11.5 reinforcers per session) relative to the lean–long condition of FCT (*M* = 7.8 reinforcers per session). Despite delivering more reinforcers per session in the dense–short condition, the total number of reinforcers delivered in the dense–short condition of FCT (69 total reinforcers) was fewer than the total number of reinforcers delivered in the lean–long condition of FCT (141 total reinforcers). The resurgence evaluation following the FCT phase showed more variable levels of resurgence of destructive behavior in the dense–short condition (*M* = 1.0 RPM) relative to the lean–long condition (*M* = 0.3 RPM). Corey's use of the FCR and the number of reinforcers delivered during the extinction challenge were similar in overall pattern to those we observed with Erica.

**Figure 5:**
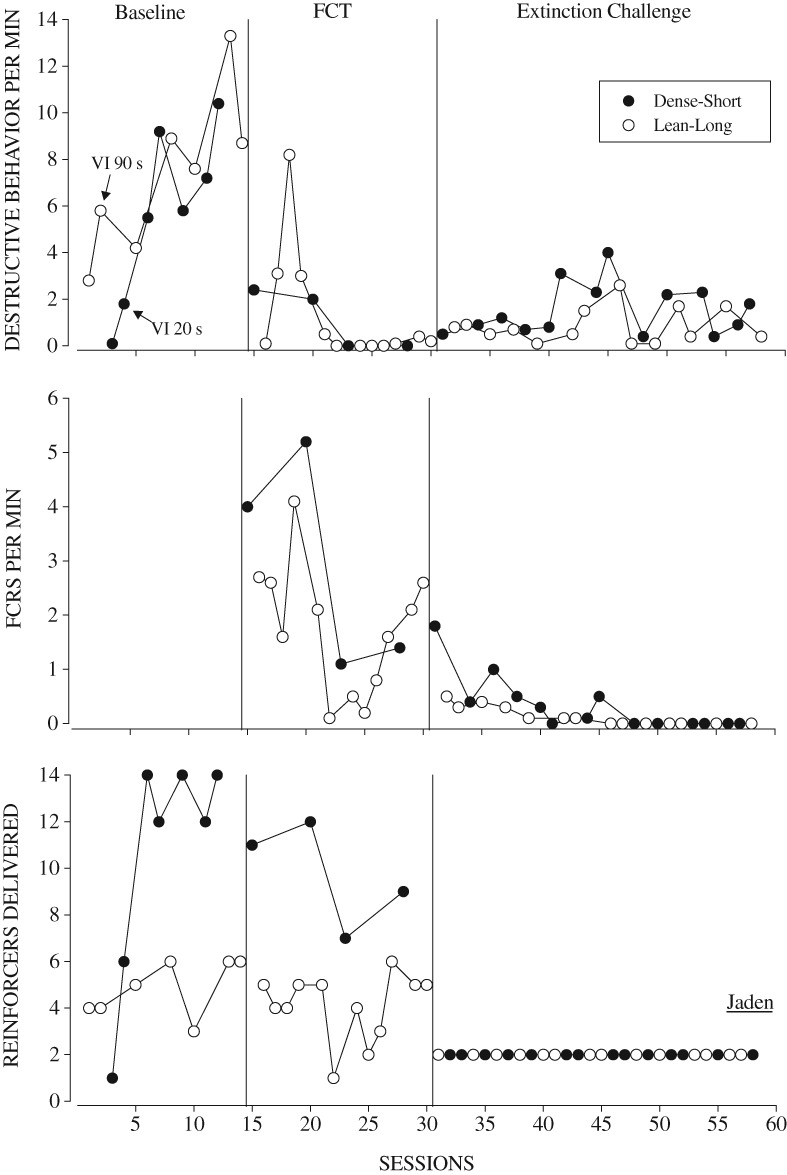
Jaden's results during the dense–short and lean–long conditions across baseline, functional communication training (FCT), and extinction phases.

Figure 5 displays Jaden's results. Jaden engaged in elevated and sharply increasing rates of destructive behavior across both conditions in baseline, with a higher rate of destructive behavior in the lean–long condition (*M* = 7.3 RPM) than in the dense–short condition (*M* = 5.7 RPM). Jaden experienced a greater number of reinforcers during the dense–short condition (*M* = 10.4 reinforcers per session) than in the lean–long condition (*M* = 4.9 reinforcers per session) in baseline. Like results for the other participants, the lean–long and dense–short conditions during the FCT phase effectively decreased Jaden's high rates of destructive behavior, with slightly higher rates of destructive behavior occurring in the lean–long condition (*M* = 1.3 RPM) relative to the dense–short condition (*M* = 1.1 RPM). Jaden emitted moderate but variable rates of the FCR across both conditions of FCT, with a slightly higher rate in the dense–short condition (*M* = 2.9 RPM) relative to the lean–long condition (*M* = 1.8 RPM). Jaden experienced a greater number of reinforcers in the dense–short condition (*M* = 9.8 reinforcers per session) relative to the lean–long condition (*M* = 4.1 reinforcers per session) of FCT. Despite delivering more reinforcers per session in the dense–short condition, the total number of reinforcers delivered in the dense–short condition of FCT (39 total reinforcers) was slightly fewer than the total number of reinforcers delivered in the lean–long condition of FCT (49 total reinforcers). The resurgence evaluation showed slightly more resurgence and relatively more variable levels of destructive behavior during the dense–short condition (*M* = 1.5 RPM) relative to the lean–long condition (*M* = 0.9 RPM). Jaden's declining use of the FCR and the constant number of reinforcers delivered during the extinction challenge were similar to the other participants.

Jaden's pattern of responding during the extinction challenge also is noteworthy in that he showed greater persistence of the FCR in the dense–short condition relative to the lean–long condition for the first five sessions of each condition (*M*s = 0.8 RPM and 0.3 RPM for the dense–short and lean–long conditions, respectively). Thereafter, FCRs remained at near‐zero rates in both conditions, and rates of destructive behavior increased in both conditions with differentially higher levels in the dense–short condition relative to the lean–long condition. That is, the data show greater persistence of FCRs during the initial sessions of the extinction challenge and greater resurgence of destructive behavior in the latter sessions of the extinction challenge, with higher rates of each response in the dense–short condition relative to the lean–long condition.

**Figure 6:**
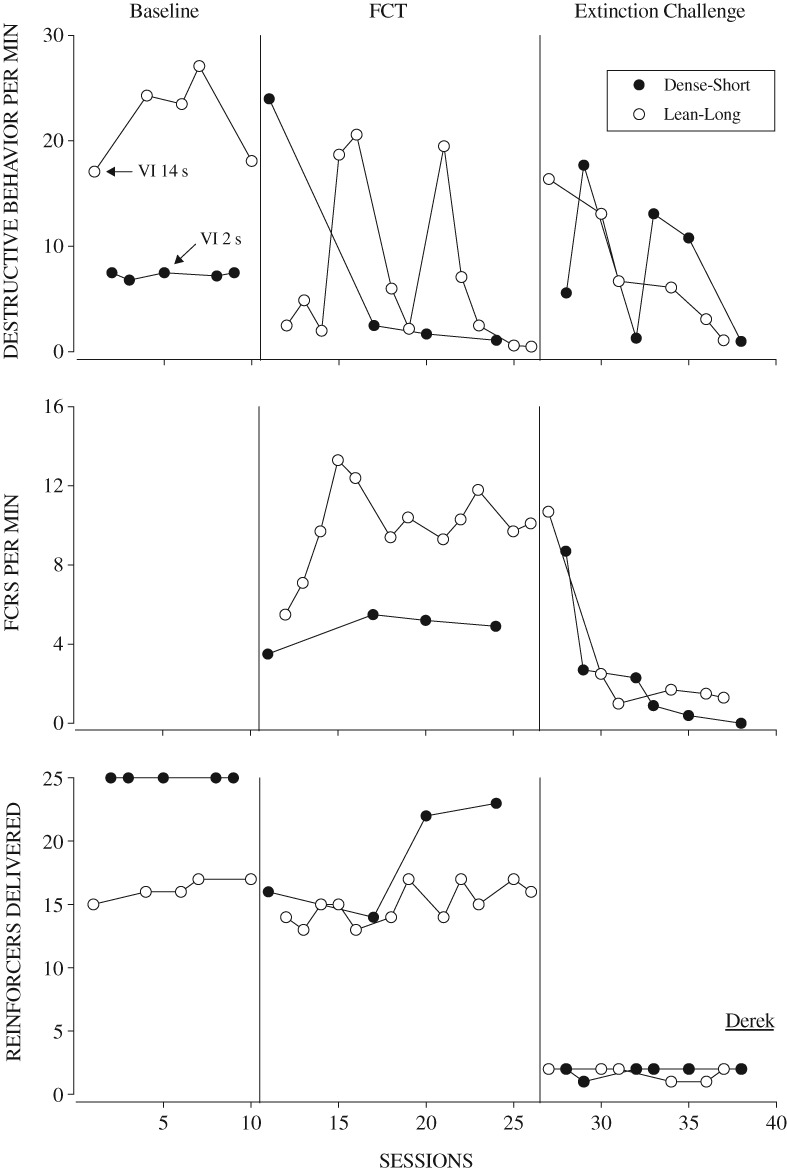
Derek's results during the dense–short and lean–long conditions across baseline, functional communication training (FCT), and extinction phases.

Figure 6 displays Derek's results. Derek engaged in elevated rates of destructive behavior across both baseline conditions, with significantly more destructive behavior occurring in the lean–long condition (*M* = 22.0 RPM) than in the dense–short condition (*M* = 7.3 RPM). Derek experienced more reinforcers during the dense–short condition (*M* = 25.0 reinforcers per session) than in the lean–long condition (*M* = 16.2 reinforcers per session) of baseline. FCT decreased Derek's high rates of destructive behavior across both the lean–long and dense–short conditions, as it did for other participants. Derek emitted high rates of the FCR during the lean–long condition (*M* = 9.9 RPM) and moderate rates of the FCR during the dense–short condition (*M* = 4.8 RPM) of FCT. Derek's destructive behavior decreased but remained variable in the lean–long condition of FCT and decreased steadily in the dense–short condition of FCT, despite both conditions producing equal average rates of destructive behavior (*Ms* = 7.3 RPM). Derek experienced a greater number of reinforcers in the dense–short condition (*M* = 18.8 reinforcers per session) relative to the lean–long condition (*M* = 15.0 reinforcers per session) of FCT. Despite delivering more reinforcers per session in the dense–short condition, the total number of reinforcers delivered in the dense–short condition of FCT (75 total reinforcers) was fewer than the total number of reinforcers delivered in the lean–long condition of FCT (180 total reinforcers). The resurgence evaluation following FCT showed slightly greater resurgence and greater variability of destructive behavior following the dense–short condition (*M* = 8.3 RPM) relative to the lean–long condition (*M* = 7.8 RPM). For all participants, use of the FCR declined across both conditions of the extinction challenge, whereas the number of reinforcer deliveries remained stable.

**Figure 7:**
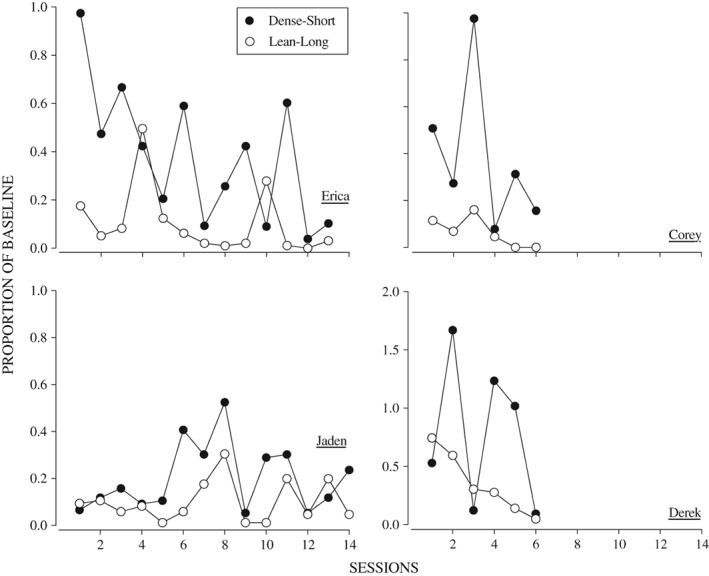
Destructive behavior during the extinction challenge expressed as a proportion of baseline responding for Erica, Corey, Jaden, and Derek.

Figure 7 displays levels of resurgence of destructive behavior during the extinction‐challenge phase expressed as a proportion of baseline levels of responding for Erica (top left panel), Corey (top right panel), Jaden (bottom left panel), and Derek (bottom right panel). Recall that in the behavioral momentum metaphor, the momentum of a response is a function of its reinforcement rate (equivalent to the mass of a moving object) times its baseline response rate (equivalent to the velocity of a moving object). By displaying destructive behavior as a proportion of its baseline rates, we control for the baseline response rates and thereby isolate the effects of reinforcement rate (cf. Mace et al., 2010; Nevin et al., 1990). We calculated proportion of baseline responding by dividing the rate of destructive behavior in each session of the extinction challenge by the average rate of destructive behavior measured over the last five baseline sessions for that condition (i.e., dense–short or lean–long).

During the extinction‐challenge phase following the lean–long condition, Erica's destructive behavior remained at low proportional rates. However, in the extinction‐challenge phase following the dense–short condition, Erica's destructive behavior persisted at higher proportional rates. The proportional rates of destructive behavior for the other three participants showed a similar pattern to Erica's proportional data, with the exception that we observed slightly less‐differentiated rates across conditions for Jaden and Derek. Across the four participants, the lean–long condition produced about two‐thirds less destructive behavior (as a proportion of baseline) relative to the dense–short condition (*M* = 65.1% lower proportional rates in the lean–long relative to the dense–short condition, range 50.2% to 83.1%).

For each participant's proportional response rates, we examined the chance probability of obtaining differences as large as the obtained differences using a randomization test (Edgington, 1967), and we also calculated Cohen's *d* effect sizes. For Erica, Corey, and Jaden, the results reached statistical significance (all *p* values < .02) and for Derek, the results approached statistical significance (*p* = .07). All effect sizes were in the large range (*M* = 1.1; range, 0.84 to 1.4; Cohen, 1988).

We programmed a tandem VT‐DRO schedule of reinforcement during the extinction challenge to decrease the discriminability of the transition from treatment to the extinction challenge and to increase overall levels of resurgence. However, delivering a lean, time‐based schedule of reinforcement during the extinction challenge may have resulted in reinstatement of destructive behavior. Thus, the recurrence of destructive behavior in our study could be attributed to resurgence, reinstatement, or to a combination of the two. We should also note that although we included the tandem VT‐DRO in the extinction challenge to increase overall levels of resurgence, some research has demonstrated that delivering time‐based reinforcers during extinction can reduce resurgence (Lieving & Lattal, 2003; Marsteller & St. Peter, 2014), and it remains possible that doing so in our study similarly decreased overall levels of resurgence. These considerations of whether to include time‐based schedules of reinforcement when testing for resurgence should be taken into account in future investigations of resurgence.

## GENERAL DISCUSSION

We tested three quantitative predictions of BMT for mitigating the resurgence of destructive behavior following successful treatment. After experiencing an FA and an initial FCT evaluation, function‐based treatment, which included extinction of destructive behavior, reduced destructive behavior to near‐zero levels for all four participants, thereby replicating prior research findings on FCT (e.g., Greer, Fisher, Saini et al., 2016). Next, we modified FCT based on BMT using Equation (1) and decreased the proportional rates of destructive behavior by about two thirds relative to the control condition. These results provide empirical support for the specific predictions for treatment modifications suggested by Equation (1).

Because our main purpose in this investigation was to test the implications of modifying function‐based, differential reinforcement interventions like FCT using Equation (1), we implemented the three primary modifications suggested by this equation simultaneously in the hopes of producing a large decrease in degree to which destructive behavior resurged when reinforcement for the FCR was discontinued; and in fact, our observed effect sizes were large by conventional standards (Cohen, 1988). Nevertheless, because we implemented these three modifications (decreased the rate of reinforcement during baseline and treatment and extended the duration of treatment) simultaneously, we are unable to determine the relative contributions of each individual treatment modification. Future investigations should examine each of the individual components separately.

Nevin et al. (2016) recently evaluated the effects of dense and lean schedules for the FCR during treatment of severe destructive behavior with FCT for four boys (ages 8 to 14) with ASD; they also conducted parallel and more extensive analyses with pigeons in another experiment within that same investigation. Results for the four boys in Nevin et al. showed similar, but perhaps less‐consistent, differences between the test (lean DRA) and the control (rich DRA) condition than we did with our test (lean–long FCT) and control (dense–short FCT). The main difference between the Nevin et al. comparison and ours is that they manipulated a single variable (DRA schedule density), and we manipulated three variables simultaneously as an intervention package (baseline schedule density, DRA schedule density, and time [number of sessions] in DRA). Another difference was that Nevin et al. signaled the availability of reinforcers during DRA using visible or auditory timers (i.e., signaled the completion of each VI component). As such, it is difficult to draw firm comparisons regarding Nevin et al.’s results and those of the current investigation.

The current findings illustrate the potential benefits of using quantitative models of behavior to identify potential modifications to function‐based treatments for destructive behavior that may not be intuitively obvious. For example, Hanley, Iwata, and McCord (2003) reviewed 277 studies that included pretreatment functional analyses of problem behavior and found that experimenters programmed dense reinforcement schedules (i.e., FR 1) in 90% of the functional analyses. Typically, researchers use the same dense schedule of reinforcement during the baselines for treatment analyses. Thus, the predictions of Equation (1) from BMT recommend the opposite of what clinicians and applied researchers typically do in standard clinical practice. Equation (1) recommends a low rate of reinforcement for destructive behavior during baseline, whereas clinicians and applied researchers typically deliver a high rate of reinforcement for destructive behavior during baseline. This represents a clear example in which the predictions of a quantitative model of behavior (e.g., BMT) lead to a potential refinement for function‐based treatments that is not intuitively obvious, one that is at odds with current “best practices.”

Similarly, Tiger, Hanley, and Bruzek (2008) reviewed 91 studies involving 204 participants treated with FCT, and in each case, the experimenters initially provided reinforcement for the FCR on a dense, FR 1 schedule. Moreover, these authors strongly recommended that behavior analysts initially deliver reinforcement for the FCR on a dense, FR 1 schedule. As is the case with baseline response rates, Equation (1) recommends a low rate of reinforcement for the FCR during treatment, just the opposite of common clinical practice and the recommendations of leading clinical researchers in the field. This represents another example in which the predictions of a quantitative model of behavior (e.g., BMT) lead to a potential refinement for function‐based treatments that is not intuitively obvious and another one that is at odds with current “best practices.” However, it is important to note that the present study provided each participant with a history of a dense schedule of reinforcement for destructive behavior and the FCR during the FA and initial FCT evaluation prior to providing a history of a relatively lean schedule of reinforcement for both responses. Thus, the effects of a lean schedule of reinforcement in the absence of a history of a dense schedule of reinforcement cannot be determined directly from our study.

The current investigation also contributes to the literature on mitigating resurgence of destructive behavior by providing two empirically based procedures for selecting lean schedules of reinforcement for baseline and FCT. Results from Nevin et al. (2016) indicate that decreasing the reinforcement rate for the FCR during treatment can mitigate resurgence of destructive behavior when alternative reinforcement is suspended. However, other studies have shown that relatively large and precipitous drops in reinforcement rate for the alternative response during DRA procedures like FCT can also result in resurgence of destructive behavior (Lieving & Lattal, 2003; Volkert et al., 2009). Thus, it may be important to develop empirical methods for identifying the lowest rate of reinforcement that maintains the FCR to reap the benefits described by Nevin et al. (2016) without evoking resurgence of destructive behavior when a lean schedule of reinforcement is introduced (Lieving & Lattal, 2003; Volkert et al., 2009).

Finally, we currently do not have any best‐practice recommendations that can provide guidance to clinicians regarding the optimal dosage of function‐based treatments for destructive behavior. One dosage question related to the problem of resurgence of destructive behavior is, “How long should we implement treatment at optimal procedural fidelity with trained therapists before introducing treatment with caregivers who may not consistently deliver reinforcement for appropriate, alternative behavior at the prescribed times?” Episodes in which caregivers do not deliver reinforcement for extended periods represent naturally occurring extinction challenges, which may result in resurgence of destructive behavior. Equation (1) predicts that longer exposures to treatment with FCT with high procedural fidelity may mitigate resurgence of destructive behavior during subsequent periods when the FCR goes unreinforced. Considering that only two levels of this factor were evaluated in the current study and in the context of other differences between conditions of different dosage (i.e., reinforcement schedule differences), future parametric evaluations of different dosages are needed to determine their impact on resurgence.

As researchers continue to investigate the conditions under which destructive behavior does and does not resurge (or relapse more broadly) and whether it does so to clinically unacceptable levels, questions regarding the risks and costs associated with any relapse‐mitigation procedure will need to be addressed. Mitigation procedures that offer the prospect of long‐term benefit will need to be weighed against any short‐term worsening in rates or severities of destructive behavior, slower acquisition of the FCR, or procedures that might otherwise delay patient discharge. Ultimately, those mitigation procedures that become widely adopted by behavior analysts will need to strike a balance between short‐ and long‐term benefit for patients and stakeholders. Our study served as a proof‐of‐concept, insofar as our objective was to evaluate whether a combination of BMT‐informed modifications to baseline and FCT mitigated resurgence. We did not evaluate whether such modifications were associated with increased risk or cost. Future research should address questions along this line.
